# Stability of the gross motor function classification system in children with cerebral palsy for two years

**DOI:** 10.1186/s12883-020-01721-4

**Published:** 2020-05-06

**Authors:** Eun-Young Park

**Affiliations:** Department of Secondary Special Education, College of Education, Jeonju University, 1200 3-ga, Hyoja-dong, Wansan-gu, Jeonju, 560-759 Republic of Korea

**Keywords:** Children with cerebral palsy, Stability, GMFCS

## Abstract

**Background:**

The prognosis of gross motor function is a major concern for therapy and intervention in children with cerebral palsy (CP). The classification system for gross motor function, the Gross Motor Function Classification System (GMFCS), is actively studied because it could be useful in the communication between professionals and families. This study aimed to verify the stability of GMFCS over 2 years in children with CP aged 2–12 years.

**Methods:**

The GMFCS level of 100 children with CP who underwent rehabilitation therapy in hospitals or who attended special elementary schools in South Korea were collected in the study. The agreements across three measurement points were analyzed in these children.

**Results:**

The weighted kappa coefficients were statistically significant (*p* < .05). The coefficients ranged from 0.690 to 0.789 in children with CP aged 2–12 years. The lowest coefficient of 0.557 was observed in children with CP aged 2–4 years between the first and third measurements points.

****Conclusions**:**

The results provided evidence of GMFCS stability for the first year and change of the GMFCS during the two-year study period in children aged 2–4 years. Moreover, the findings indicate that the stability of GMFCS varies with time, duration, and age. It is recommended that GMFCS assessments be performed periodically, which are even more necessary for children with CP aged 2–4 years.

## Background

The Gross Motor Function Classification System (GMFCS) is a five-level evidence-based tool that measures the gross motor function of children with cerebral palsy (CP). The gross motor functions that are emphasized in GMFCS are sitting, walking, and wheelchair mobility. The GMFCS level that is determined does not depend on what is known to be routinely possible at home, school, or in the community settings; that is, it is rather an indication of what they can do better than what they actually do [1]. The classification system groups data and subjects according to common characteristics, which results in a reduced number of data. The usefulness of a classification system depends on how easily and clearly the classification scheme is described and how it can be categorized at significantly different levels. The classification system is meant to classify and categorize rather than evaluate [2].

GMFCS is rapidly being accepted in clinical practice and research [3], and has been reported to be directly related to the limitations in activity and participation [4]. A previous classification system, the Swedish classification (SC) of clinical CP subtypes [5], could not provide information on the child’s functional abilities in everyday life; however, the GMFCS could provide this information, and it was used in the clinical setting for communication between specialists and other persons [2]. The usefulness of a classification system depends on how easily and clearly the classification scheme is described and how it can be categorized at significantly different levels. The classification system is meant to classify and categorize rather than to evaluate [2].

Information on the current functional state of children with CP can help provide an adequate quality of life for them and their families, ensuring a promising future [6, 7]. Specifically, information on how gross motor function of children with CP develops over time and its stability helps clinicians in planning the treatment strategy. Insights related to changes in gross motor function of children with CP could be useful to develop programs to prepare for adolescence and adulthood [8].

The GMFCS levels have been proved to be important determinants of functioning in daily activities and social participation in individuals with CP [9]. Therefore, prediction of the GMFCS level can play an important role in predicting future functional changes of the child. An important issue in decision-making and parental counseling is whether children with CP can maintain the same level of competence or be reclassified to different levels over time [10]. Moreover, without a clear understanding of the natural history of motor development in CP, it is difficult to assess the impact of intervention beyond improvements in motor function that may have been due to normal growth and development [11]. However, there is not enough information yet about the natural change, as opposed to stability, in children with CP.

Stability of GMFCS in children with CP has been reported. However, there are some concerns about the data in previous studies. One study used data from two different therapists in the same children [12]; there was no information about whether the therapist was the same for the two measurements [10]. Some studies used chart data [13]. Although there was a high inter-rater reliability between the therapists [11], the results from different therapists’ assessments and from parents might differ. For this reason, this study used data from the same therapists for verifying the stability of GMFCS. There is more information regarding stability of GMFCS in children with CP across different age groups, especially those aged 2 to 4 [10]. The GMFCS levels that are determined around the age of 12 are highly predictive of adult motor function [13]. Previous studies on stability of GMFCS in children with CP have concluded that stability would be higher for children aged 4 years or older than for children younger than 4 years [14]. After reviewing the previous studies, this study was conducted to examine the stability of GMFCS in children aged from 2 to 12 years. This study aimed to investigate the stability of GMFCS in children with CP aged < 12 years through three measurement points over 2 years. The specific research purpose was that the change in GMFCS levels over the 2 years can be investigated either through looking at the agreements.

## Methods

### Study participants

The study was approved by the ethical review board of Jeonju University in South Korea. All parents of children with CP provided written consent for their participation in the study. The study participants were 100 children with CP who underwent rehabilitation therapy in hospitals or attended special classes in elementary schools across South Korea. They were diagnosed by physicians. The researcher initially contacted hospital staff and teachers in elementary schools to recruit the target population and then identified parents who were willing to participate. A total of 10 hospitals and 2 schools agreed to recruit participants. Initially, 105 participants were recruited and 100 were included in the study. Inclusion criteria were as follows: age between 2 and 12 years, diagnosis of CP from a doctor, and provision of consent for the study. Exclusion criteria were as follows: had selective dorsal rhizotomy, with other neuromotor disabilities, and with other musculoskeletal or nerve abnormalities. Mean age was 7.72 years (standard deviation = 3.28 years). The general characteristics of study participants are presented in Table 1.

**Table 1:** Characteristics of participants

Characteristic	% (n)
Gender	
Male	61.0 (61)
Female	39.0 (39)
Type of cerebral palsy	
Spastic	76.0 (76)
Dyskinetic	9.0 (9)
Hypotonic	10.0 (10)
Ataxic	5.0 (5)
Distribution of motor impairment	
Quadriplegia	40.0 (40)
Triplegia	3.0 (3)
Diplegia	44.0 (44)
Hemiplegia	13.0 (13)

### GMFCS

The GMFCS, which describes the gross motor function level in children with CP, was used to evaluate the gross motor functional level of each child. GMFCS consisted of five levels based on ambulatory function [15]. Initial GMFCS was designed to be used in children aged 2–12 years [16]. The inter-rater reliability of GMFCS measures was reported to be 0.84 [16]. Excellent agreement of the GMFCS was reported in children aged 2–12 years (kappa = 0.75) [1].

### Procedure

The present study was a secondary analysis of the data from a large longitudinal study approved by the Research Ethics Board of Jeonju University (Jeonju University IRB-1). Data regarding GMFCS were collected by physical therapists (PT). The GMFCS level of children with CP was classified by his/her own PT who had treated children with CP for more than 6 months. The PT determined the GMFCS level through observation. The same therapists evaluated the same child for all three ratings and were blinded to the previous scores. The total number of measurement points was three over 2 years. The interval between measurements was 12 months. Authors requested the PT for the data every 12 months.

### Data analysis

Children with CP were grouped based on their age. Group 1 was aged from 2 to 4 years, group 2 was aged from 4 to 6, and group 3 was aged from 6 to 12 years. Two statistical methods were used in this study. One was descriptive statistics for verifying the characteristics of the participants and calculating the change rate in the GMFCS level across three measurement points. Another was the weighted kappa coefficient used for verifying the agreement rate in GMFCS levels over 2 years. The weighted kappa values were 0 to 0.20 for slight agreement, 0.21 to 0.40 for fair agreement, 0.41 to 0.60 for moderate agreement, 0.61 to 0.80 for substantial agreement, and 0.81 to 1.00 for almost perfect agreement [17]. Weighted kappa coefficient was used to investigate the change in the agreement of GMFCS levels across the three measurements. The level of significance was .05. The SPSS version 23.0 software (SPSS Inc., Chicago, IL, USA) was used for the statistical analysis.

## Results

### Agreement across three measurement points

In terms of the GMFCS levels’ stability, the weighted kappa coefficients were 0.690 to 0.783 (Table 2). The lowest coefficient was between the first and third measurement points, whereas the highest coefficient was between the second and third. The lowest coefficient between the first and third points and the highest coefficient between the second and third of weighted kappa were observed to have the same pattern in all age groups except for the 2–4 years age group. The coefficient was lowest between the first and third measurement points in children aged 2–4 years.

**Table 2:** The weighted kappa across three measurement points

Age Range	Category	Weighted Kappa	Lower 95% CI	Upper 95% CI
2 ~ 12 years (*n* = 100)	First and second	0.763^*^	0.675	0.850
	Second and third	0.783^*^	0.715	0.852
	First and third	0.690^*^	0.589	0.791
2 ~ 4 years (*n* = 15)	First and second	0.754^*^	0.570	0.939
	Second and third	0.719^*^	0.530	0.908
	First and third	0.557^*^	0.301	0.812
4 ~ 6 years (*n* = 25)	First and second	0.715^*^	0.440	0.991
	Second and third	0.785^*^	0.596	0.973
	First and third	0.652^*^	0.358	0.945
6 ~ 12 years (*n* = 60)	First and second	0.757^*^	0.645	0.870
	Second and third	0.778^*^	0.689	0.868
	First and third	0.721^*^	0.602	0.839

*Note.* The superscripted asterisk indicates significant difference (*p* < 0.05)

**Fig. 1:**
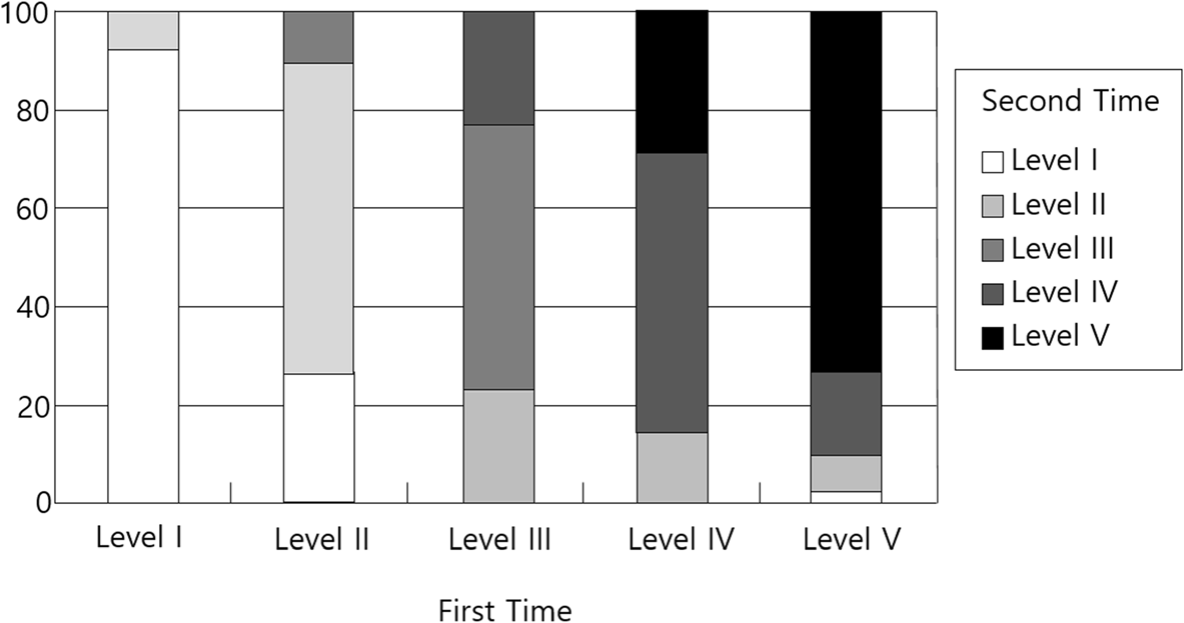
The change rate between the first and second measurements

**Fig. 2:**
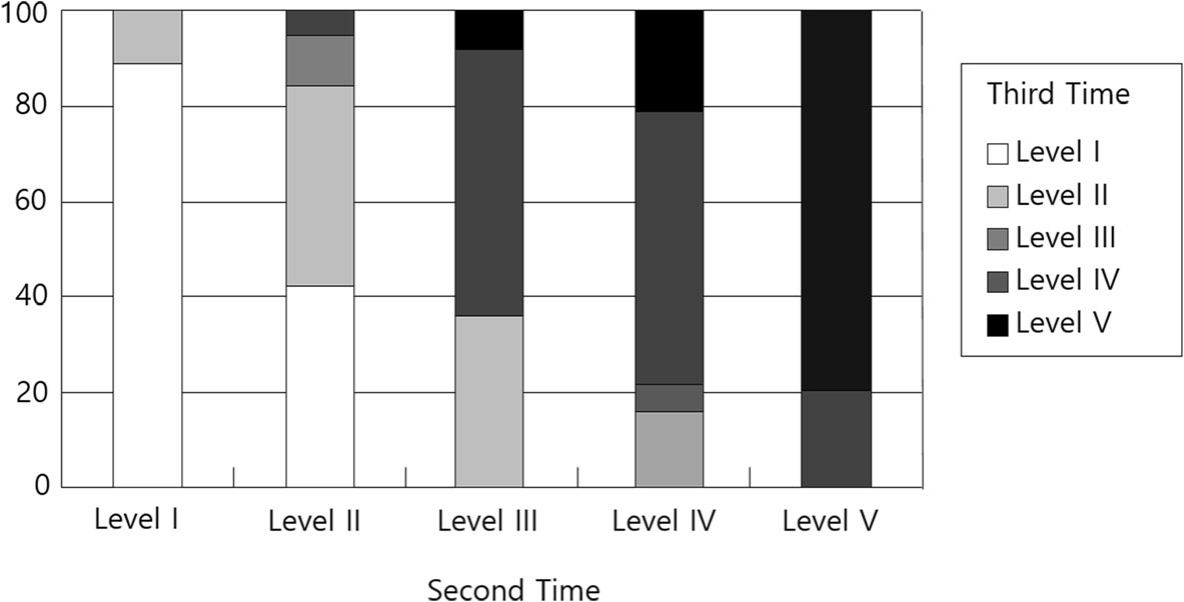
The change rate between the second and third measurements

**Fig. 3:**
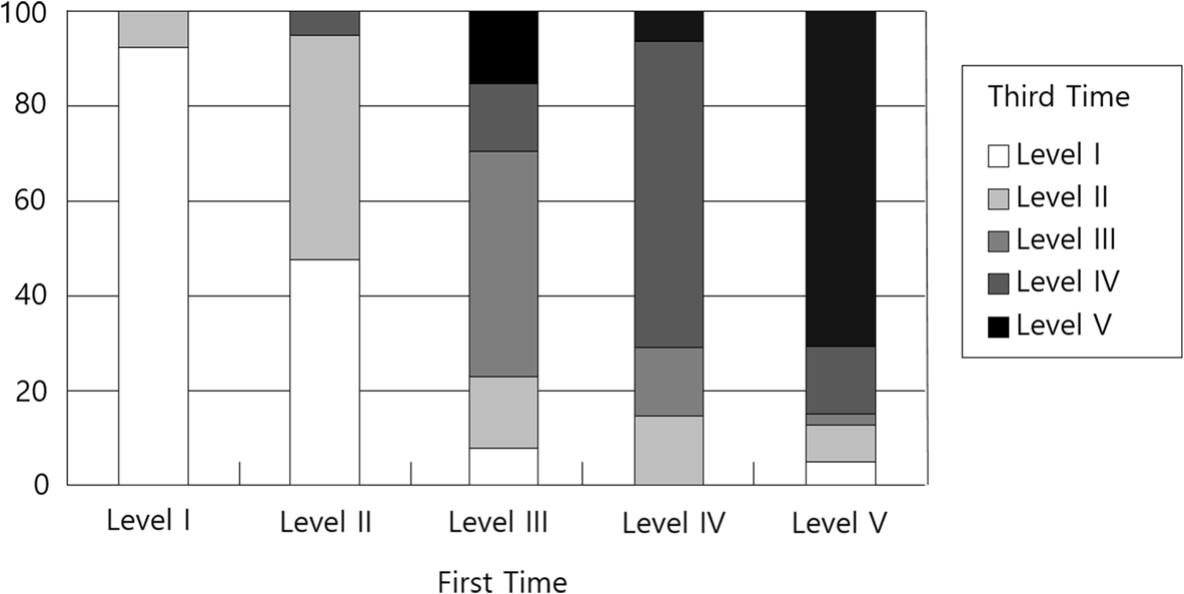
The change rate between the first and third measurements

As shown in Table 3, of the 100 participants, 69 remained at the same level for 1 year when the first and second measurements were performed. Of the 31 that showed change, 10 had higher GMFCS levels and 21 had lower GMFMS level. Only one change in level (e.g., from level I to level II) was shown in all children who went to a higher level. The change ratio is presented in Fig. 1, Fig. 2, and Fig. 3. The largest rate of change was shown in level III (46.2%), followed by level IV (42.9%). The GMFCS level in 67 children with CP remained the same, whereas it changed in 33 children with CP between the second and third measurement time. Ten had a higher GMFCS level in the third measurement, whereas the others had lower levels than the level at the second measurement point. Of the 100 participants, 65 remained at the same level for 2 years between the first and third measurements. Of the 35 that showed change, seven had higher GMFCS levels in the third measurement time from the first measurement time, and the remaining 28 children with CP had lower GMFCS levels. The change rate was 7.7% in level I, 52.6% in level II, 53.9% in level III, 35.7% in level IV, and 29.3% in level V.

**Table 3:** Change of the GMFCS levels

Category	No change	Go to higher level	Go to lower level
From first to second time	69	10	21
From second to third time	67	10	23
From first to third time	65	7	28

*GMFCS* Gross Motor Function Classification System

## Discussion

This study aimed to determine whether the GMFCS level of children with CP aged between 2 and 12 years was stable over 2 years. First, the weighted kappa across the three measurements points was calculated. The weighted kappa coefficient was 0.61 to 0.80, indicating a substantial agreement, and 0.81 to 1.00, suggesting an almost perfect agreement [17]. The weighted kappa was the highest between the second and third measurements (0.783) and the lowest between the first and third measurements (0.690) in children aged 2–12 years. The lowest coefficient was 0.557 in children aged 2–4 years between the first and third measurements. McCormic et al. [13] reported that the weighted kappa was 0.895 in the 103 participants aged 17–38 years. Palisano et al. [10] reported that the weighted kappa coefficient between the first and last measurements was 0.84 and 0.89 for children < 6 years old and at least 6 years old, respectively. The lower weighted kappa coefficients in this study might be due to the initial GMFCS level of children. In Palisano et al.’s study [10], 45.9% children with CP below 6 years old and 48.2% above 6 years old with GMFCS level I and V participated. The change of GMFCS level was shown in GMFCS level II to IV in this study and previous studies. Further studies about change or stability of GMFCS level should interpret the agreement results considering the initial level. Moreover, the stability according to GMFCS level could be completed in further studies. The results of this study showed that follow-up evaluation should be conducted in GMFCS level II to IV.

The results of weighted kappa in this study indicate that the change of GMFCS level between the first and third years of the two-year study period is larger than that between the first and second years, and between the second and third years. The results of this study, which showed a high rate of re-evaluation at a higher GMFCS level, are similar to those of a previous study, which suggests a change in the gross motor function in children with CP. Gross functional ability of children with CP decreases when they get older. Jahnsen et al. [18] revealed that 45% of children with CP showed a decline in ambulation as they progressed to adulthood, whereas 27% showed an increase in ambulation. A prognosis study based on a large population record reported that the gross motor curves appear to reach plateaus by about age 7 [15]. However, the change in direction in this study was different from that in the previous study that reported the plateaus and a decline. The possible reason for increasing gross motor function in children with CP who participated in this study was the different age range.

Second, the change ratio across three measurement points was provided. Our study results demonstrated a higher change rate in GMFCS level. The 73% agreement in children with CP born between 1990 and 2007 was reported by a recent study in 2017 [19], which included 7922 assessments. The original CanChild study of GMFCS stability had a higher agreement of 76 and 83% for children younger and older than 6 years, respectively [10]. Direct comparison might be difficult because this present study was different from the previous studies in terms of the study period, frequency of measurement, and assessor. In Palisano et al.’s study [10], the mean study period was 33.5 months, ranging from 6 to 52 months, and the GMFCS ratings were obtained by different therapists. In Alriksson-Schmidt et al.’s study [19], only 11.6% of the participants had the same physical therapist at all assessments. Moreover, their study period was 2 years, and the ratings were obtained by the same therapist. The frequency of measurement in previous studies was different in each child with CP. Palisano et al.’s study [10]‘s varied from 2 to 7 and measurement was completed every 6 months for children less than 6 years old and every 9 to 12 months for children who were at least 6 years old. Alriksson-Schmidt et al.’s study [19] reported 11 median number of GMFCS ratings during 7 years. Another difference between this study and previous studies was due to the source of the data. Gorter et al. [20] verified the stability of the GMFCS in 77 infants. However, this study on the stability of the GMFCS, with the exception of a few, involved through a chart review.

Although slightly lower than those of previous studies that showed stable GMFCS levels in children aged 2–12 years, the results of this study also showed stability of the GMFCS levels. This indicates that the proportion of children whose level did not change was higher than that of children whose level changed, and the change was higher in age range from 2 to 4 years. The results of this study suggest that it is likely that the GMFCS levels measured during childhood will not be maintained until adulthood, and regular re-evaluation of the GMFCS levels would be necessary.

The present study results may suggest a need to re-rate the participants every 2 years, although this study did not provide periodic reevaluation results. Insight regarding aging-related effects on gross motor function in children with CP might be used to develop policies and programs to prepare such children for adulthood.

This study has some limitations. The specific limitations and recommendations of a future research were followed. First, physical therapists were not aware of the previously measured scores, and the measurements were performed at 12-month intervals, but the therapists may have been aware of the previous measurement scores because they assessed the same child. This could affect the results. Second, no data for adolescents were provided in this study. On the basis of the report of deteriorating gross motor function after adolescents with CP become adults [18], comparing the change in GMFCS level between children and adolescents will be necessary to provide useful information on the future progress of gross motor function. Third, we reported the increase and decrease in GMFCS level in children with CP; however, the results lack specificity. Especially, the cause of large change, such as the change from level I to V, needs to be investigated although the number of cases was small. These large-change cases were also reported in the previous study but the specific reason for this could not be found [10]. In future studies, will be necessary to investigate the functional changes of children with CP and to identify the variables that affect these changes. Understanding why children’s GMFCS levels are changing can help in their prognosis. Moreover, the information on how the gross motor function of children with CP develops over time helps clinicians in planning the treatment strategy.

## Conclusions

This study shows evidence that the stability ratio of the GMFCS was high and that the change ratio also existed in children with CP aged between 2 and 12 years. The GMFCS level change was large in level III and IV during the first year and change of GMFCS level II was larger over 2 years. The long study period could likely lead to GMFCS level change, especially in younger children with CP aged 2–4 years. Moreover, the findings indicate that the stability of GMFCS varies with time, duration, and age. A periodic assessment of GMFCS is needed, which is even more necessary for children with CP aged 2 to 4 years.

## Data Availability

The datasets used and/or analyzed during the current study are available from the corresponding author on reasonable request.

## Abbreviations

CP: cerebral palsy
GMFCS: Gross Motor Function Classification System
